# Posttraumatic stress disorder: An exploratory study examining rates of trauma and PTSD and its effect on client outcomes in community mental health

**DOI:** 10.1186/1471-244X-5-21

**Published:** 2005-04-26

**Authors:** Irene M Howgego, Cathy Owen, Lenore Meldrum, Peter Yellowlees, Frances Dark, Ruth Parslow

**Affiliations:** 1 Academic Unit of Psychological Medicine, Australian National University The Canberra Hospital, Australian Capital Territory 2605, Australia; 2 Medical Education Unit, Australian National University, Australian Capital Territory, 0200, Australia; 3 PO Box 198 Kenilworth Queensland, 4574, Australia; 4 Centre for Health Teaching, University of California, Davis, CA 95616, United States of America; 5 West End Mental Health Service Brisbane, Queensland, 4101, Australia; 6 Center for Mental Health Research, Australian National University, Australian Capital Territory, 0200, Australia; 7 Formerly at University of Queensland ST LUCIA Queensland 4072 Australia

## Abstract

**Background:**

Rates of trauma and Posttraumatic Stress Disorder (PTSD) were examined in order to compare the profile in clients of an Australian Public Mental Health Service with that reported in the international literature for clients with major mental illness and to explore the effect of this on client health outcomes. Potential factors contributing to increased levels of trauma/PTSD in this group of clients and the issue of causality between PTSD and subsequent mental illness was also explored.

**Methods:**

A convenience sample of 29 clients was screened for trauma and PTSD using the Posttraumatic Stress Diagnostic Scale™ (PDS) and selected outcome measures. Paired and independent samples t-test and ANOVA were applied to the data.

**Results:**

High levels of undocumented trauma and PTSD were found. Twenty clients, (74%) reported exposure to multiple traumatic events; 33.3% (9) met *DSM IV *diagnostic criteria for PTSD. Significant difference was found for PTSD symptomatology, severity and impairment and for client and clinician-rated scores of Quality of Life (QOL) outcomes in the PTSD group. No effect for PTSD symptomatology on the Working Alliance (WA) was found. Factors that may influence higher rates of PTSD in this group were identified and included issues associated with the population studied, the predominance of assaultive violence found, and vulnerability and risks factors associated with re-traumatisation within the social and treating environments.

**Conclusion:**

A similar trauma and PTSD profile to that reported in the international literature, including greater levels of trauma and PTSD and a poorer QOL, was found in this small sample of clients. It is postulated that the increased levels of trauma/PTSD as reported for persons with major mental illness, including those found in the current study, are primarily related to the characteristics of the population that access public mainstream psychiatric services and that these factors have specific implications for service delivery, and raise issues of efficiency and effectiveness of resource use in achieving successful outcomes in public mental health services for clients with co-morbid PTSD. Further research with a more rigorous design is needed to test these preliminary findings within Australian Community Mental Health Services.

## Background

PTSD is emerging as a major public health problem worldwide [1]. Recent epidemiological studies within Australia [2] and America [3] identified rates of PTSD within the general population as 1.3% *(DSM IV criteria) *3.3% (ICD 10 criteria) and 7.8% *(DSM-III-R criteria) *respectively. Whilst this growing recognition of the prevalence of PTSD is stimulating research, activity in the broad spectrum of psychiatry is still limited. Despite the knowledge that high rates of trauma are associated with persons with mental illness few studies have examined trauma and PTSD in this population [4-6], and the majority of these stem from the United States of America [7]. PTSD research in mainstream psychiatry clearly highlights the complexity of trauma/PTSD in persons with a co-existing psychiatric illness [6,8]. The emerging profile showed a group of respondents with high levels of previously undetected trauma (51% – 98%) [6,9] and PTSD (22.2% – 66%) [9,10] who exhibited the phenomena of multiple traumatisation [5,6,10-14]. The primary type of trauma identified was interpersonal in nature (physical and sexual assault) and included both childhood and adulthood victimisation. The general lack of recognition and documentation of trauma and PTSD evidenced in a number of studies [4,6,9,11,12,14,15] coupled with the high rates of trauma and PTSD found, reflects the general consensus in the psychiatric literature that the problem is under diagnosed and potentially untreated in this population.

Whilst existing studies provide valuable data and insight into PTSD in persons with another mental illness, more needs to be done to determine the applicability of these findings outside of the American context. Australian PTSD research in conventional psychiatry is in its infancy with only one study to date reporting on PTSD in a psychiatric in-patient unit [4]. This showed clients with an undocumented trauma rate of 61% and PTSD rate of 28% based on *DSM-III-R *criteria [4]. PTSD was also found to be the *incident disorder *in 50% of clients and preceded major depression in 83% of these clients (n = 141).

Despite these findings, routine assessment of trauma (and therefore, diagnosis of PTSD) in persons presenting to Community Mental Health Services (CMHS) is often "overlooked" in the absence of PTSD symptomatology as the presenting complaint [15]. This has critical implications for clinical management as clients generally will not volunteer this information either from a reluctance to re-visit the trauma, fear of clinician response or simply not recognising the relevance of any prior trauma to their current problem [16]. Consequently, nationally and internationally, the recognition, diagnosis and treatment of PTSD in clients with comorbid psychiatric diagnoses is at best sporadic and poorly understood by the majority of clinicians [1,17-21]. Compounding this is the fact that conventional psychiatry and mental health service delivery in Australia has been slow to embrace concepts from the field of traumatic stress [17]. Leo Sher, [21] in correspondence published in an Australian psychiatric journal, concluded that "there is a pressing need to improve recognition and treatment of PTSD".

### Implications for mental health services and client outcomes

These findings from the literature have important implications for successful outcomes in Mental Health for the individual, the clinician, the service provider and society in general. On the individual level PTSD is known (and acknowledged) to be co-morbid with a variety of other psychiatric disorders particularly mood and anxiety based problems [3,4,7,15,16,19]. These disorders represent the 'core business' of CMHS. Therefore, the potential exists for clients being treated in such services for any of these disorders, to have underlying PTSD symptomatology.

Untreated co-morbid PTSD in persons with another major mental illness is associated with important negative effects such as increased symptom severity for both diagnoses, increased hospitalisation, prolonged treatment and poorer overall health outcomes for the individual [4,6,8,16,19,22]. Additionally, the therapeutic alliance between client and clinician may also be compromised, further eroding the likelihood of achieving positive client outcomes [6]. This latter factor is of key clinical importance as the role of the relationship in achieving positive outcomes is well documented [23-26]. For the service provider, all of the above result in greater overall treatment and management costs and sub-optimal resource use. Ultimately, society bears both the social and financial cost of PTSD. This burden to both individuals and society is acknowledged by contemporary researchers [7,16] leading to the conclusion that PTSD is one of the most serious and disabling psychiatric disorders [1].

There are no clear causal links between PTSD and other mental illness. The contemporary literature discusses the interplay of risk factors such as personal and family psychiatric history, gender, ethnicity, and type of trauma [22,27] and hypothesises on causal pathways and co-morbidity with other Axis I diagnoses including psychotic-based illnesses [16]. However, the exact relationship between trauma/PTSD and mental illness, how this effect is mediated, and whether it differs diagnostically across the spectrum of mental disorders is poorly understood and requires greater research [8,28].

**Figure 1:**
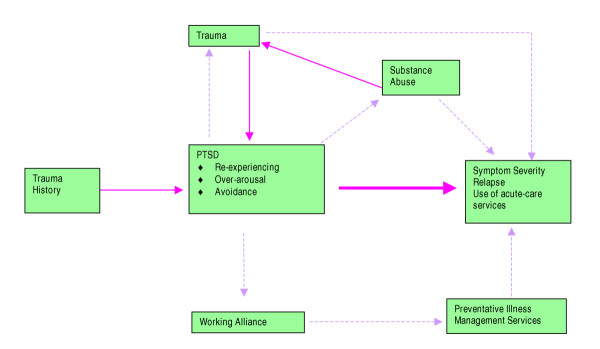
Interactive model of Trauma, PTSD and Severe Mental Illness [8] used with permission.

A model of Trauma and Mental Illness (Figure 1) proposed by Mueser et al. [8] demonstrates the potential for explanation and examination of these complex interactions. Within the context of this model, it is theorised that PTSD symptomatology plays a central role in the severity and course of mental illness through two mechanisms. First, directly from the 3 symptomatology clusters of *Re-experiencing, Avoidance*, and *Hyper-arousal *either collectively or individually (represented by the solid lines). Second, indirectly (represented by broken lines) through the effects of correlates of PTSD such as substance abuse and re-traumatisation, which further compromises the course and severity of symptoms. Additionally, the potential for a poor working alliance with clinicians resulting from all these factors, may lead to the patient receiving fewer preventative illness management services such as case management and/or medication management, thereby further compromising their status [8].

The following research questions arising from the literature review were explored in this study:

1. What was the trauma and PTSD profile for clients in an Australian CMHS?

2. How did this profile compare to that reported in the international literature for similar groups of clients?

3. Did the treating health professional know of this profile?

4. What impact did PTSD symptomatology have on

a. The Working Alliance between clients and their case managers

b. Client health outcomes?

5. What factors may be contributing to the higher levels of trauma and PTSD reported in the literature for this population?

6. What are the possible links between PTSD and other mental illness?

This paper discusses the exploratory results from a small (n = 27) sample of clients with predominantly Axis I diagnoses who were screened for trauma/PTSD at baseline (T1). All clients were receiving case management services for their primary disorder within a public-funded CMHS.

## Methods

### Design

The primary study used a prospective time-series design. Data was collected at baseline and planned for five time points (baseline screening and selected outcome measures at 1 month, WA baseline and the remaining outcome measures at 3 months with repeat of all measures at 6 months). However, this was reduced to three time-points due to client retention problems. The study was funded by the National Health and Medical Research Council (NHMRC) and was a joint project between the University of Queensland and Mental Health ACT (MHACT). Ethical approval was obtained from both institutions.

### Setting

The research initially commenced in Brisbane, Australia and was re-located to the Australian Capitol Territory (ACT) in the early phase of implementation. MHACT serves a population of approximately 320,000 residents, 21% of whom experienced a mental health disorder compared to the national average of 18% [29].

The ACT research site is an integrated Public Mental Health Service. As such, the service structure included comprehensive community and hospital-based elements that provided preventative, acute and ongoing care, case management and rehabilitation services integrated within a single agency (including a shared filing system) in order to facilitate continuity of care and ease of access for clients to the various service elements. Other elements included a GP Liaison and Court Liaison service. Specialised PTSD services did not exist within the service. Four geographically diverse adult CMHS located throughout the ACT community were used as study sites.

Case management services included the provision and/or co-ordination of a wide range of agency and external services that targeted the client's primary psychiatric diagnosis and were tailored to individual client needs. Services included medical management, medication monitoring, rehabilitation, counselling services, group therapy, such as depression, anxiety, anger management, leisure/social activities, accommodation support, drug and alcohol services, financial management, legal services/representation and respite care and hospitalisation as needed.

### Sample

#### Eligibility Criteria

Adult clients who were entering a 'new' therapeutic relationship with a case manager (CM) and were expected to receive case management service for 12–18 months in one of the four adult community mental health teams were eligible to participate. A 'new therapeutic relationship' was defined as that of a client who was either entirely new to the service or one who was being re-assigned to a new CM. No diagnostic restriction was placed on participants. However, the ability to respond to written and spoken English was essential; interpreter services were considered, however this was rejected as a threat to validity. Only one potential client was excluded on the grounds of being non-English-speaking.

### Sample selection and procedures

#### Pre-Recruitment

Prior to the recruitment phase a series of information sessions detailing the aims and purpose of the research together with the recruitment and selection procedures were given for the CM's at each of the study sites.

#### Recruitment

A researcher (IH) attended weekly team allocation meetings (assignment of new clients to CM's) in an effort to aid recruitment, address any concerns regarding the study and generally facilitate the process. Recruitment occurred over a 9-month period.

#### Selection

Potential participants were selected consecutively from a random start date using the referral list to the teams. The CM initially approached clients about the study. Eighty-four clients from a pool of 211 met the selection criteria, 27 of these consented to participate in the study, a recruitment rate of 34.5%. Although case managers nominated a total of 211 client names, 74 of these did not meet the selection criteria, primarily the criterion relating to the term of case management. Clinical concerns expressed by the CM 's re the client's mental state excluded another 13 clients (CM's considered that approaching the client about the research would have an adverse impact on either the client's current mental state or the developing therapeutic relationship). As the relationship was the primary independent variable examined in the larger study, it was essential to have both CM and clients engaged in the study, therefore CM clinical judgments regarding the clients well-being were respected. Thirteen clients failed to engage with the service. Time from the initial contact (1 month) was critical to baseline data collection (relative to the interaction with WA) and 27 clients had not been approached by their CM regarding the research in this time period and were, therefore, lost' to the study.

#### Procedures

An information leaflet was given to interested clients (by their CM) and an appointment made for a researcher to follow-up on the written material. Explanations of the purpose of the study, issues of confidentiality and consent procedures were discussed with clients. Written consent was obtained from participants. Interviews were conducted by a research officer (I. H), and clients were paid $10 for each completed interview. The final sample size was twenty-nine patient-case manager dyads.

### Instruments

#### Trauma/PTSD

Posttraumatic Stress Diagnostic Scale™ (PDS) [30]; a 49 item self-report scale based on the *DSM IV*[31] PTSD diagnostic criteria A-F.

The PDS assesses current PTSD symptomatology as experienced by the client **1 month **prior to interview. The possible number of endorsed symptoms is 0–17 and the Symptom Severity score 0–51. Impairment is measured over 9 Functional Life Areas and defined as 0= No Impairment, 1–2 areas = Mild Impairment, 3–6 areas = Moderate Impairment, and 7–9 areas = Severe Impairment.

Although the scale was designed for self-completion, it was administered via interview to maintain internal validity by ensuring clients were responding to items measuring PTSD symptomatology and not potential overlap symptoms from their primary diagnosis. The interview was re- focused on the reported trauma as required.

Clients who identified multiple traumas were asked to nominate the 'one that bothered them the most' consistent with the administration requirements of the instrument. The PDS has demonstrated validity and reliability and is recommended as particularly useful when used for screening and assessing PTSD, especially in 'at risk' populations in clinical and research settings [30].

The CM's knowledge of the client's trauma profile was also sought and they were asked to complete Parts 1 and 4 of the PDS. Part 1 listed the traumatic event. CM's were asked to respond to this and indicate any event/s that they knew the client had experienced. Part 4 lists nine life areas that may be impaired as a result of the trauma. If CM's answered 'yes' to any trauma in Part 1, they were asked to respond to this and indicate if they knew whether the client's life had been effected by the trauma in any or all of the areas listed. Additionally, CM's were asked, if to their knowledge, the client was receiving any treatment for the trauma.

**The Working Alliance Inventory-Client Form (WAI-C) **[32]; a 36-item questionnaire that assesses the client's thoughts and feelings about their relationship with their clinician through the three principal components of the Working Alliance: Tasks, Goals and Bonds. The form is available in both self-completion and interview format, the latter was used in this study.

**The Working Alliance Inventory – Case Manager Form (WAI-CM) **[32]; a parallel measure of the client form that assesses the clinician's thoughts and feelings regarding their relationship with the client. Both these measures have demonstrated validity and reliability [32]. Permission was obtained from the scale designer to modify some wording to better reflect the clinical context of the research, that is, clinical case management as opposed to psychotherapy/psychology.

**Quality of life, symptomatology and general functioning: **these key outcomes were measured using the following:

**The Australian World Health Organization Quality of Life-BREF (WHOQOL-BREF) **[33]. Based on subjective measures of quality of life only, it consists of 24 items in 4 domains – Physical Health, Psychological Health, Social Relationships and Environment. Two items also measure overall quality of life and general health. Validity and reliability have been established [33]. CM's completed a modified version that reflected their perception regarding the client's Quality of Life (QOL). CM's were not asked to rate this from the client's perspective but as a clinical judgment of the client's QOL.

**Health of the Nation Outcome Scale – Version 4 **[34]; a clinician-rated measure of client health outcomes and useful for measuring improvement in client status over time; it is a 12-item scale with four sub-scales representing different problem areas of Behaviour, Impairment, Social Functioning and Symptom domains. Reasonable reliability and validity has been established within Australia and the UK [35,36]. Clients also completed the 'Health Questionnaire', the client equivalent of the HoNOS. Although this instrument is not in the formal suite of HoNOS measures, (as it did not progress past preliminary field trials) after consultation with the HoNOS UK centre, it was decided to use it in this study and attempt validation of the measure. The results of this validation will be presented elsewhere.

**The Abbreviated Life Skills Profile (LSP-16) **[37,38]; a 16-item scale developed from the original Life Skills Profile. Reliability and validity have been established.

This measure together with the HoNOS is recommended for use by service providers as part of outcome measurement initiatives in the Australian National Mental Health Strategy [38].

#### Administration

A major aim of the larger study was to gain both client and CM perspectives; therefore, with the exception of the LSP-16 all measures had both client and CM versions. Where these did not already exist, permission was sought from the scale designer for any modifications undertaken.

#### Client measures

All client measures except the Health Questionnaire were by face-to-face interview that was conducted by a single researcher (IH). The Health Questionnaire was originally designed for self-completion and was administered in this mode as part of the validation process.

#### CM measures

these were presented as a suite of questionnaires in booklet form and provided to the CM prior to the client interview. CM's were notified of the date of the interview in writing and were requested to complete the questionnaires within a maximum 5 day time frame either side of the client interview, that is, 10 days in total, in order to improve reliability and validity of clinician and client score comparisons.

#### Preparation

A series of familiarity sessions, which included a sample suite of client and clinician measures, and a 'hot-line' contact for any subsequent queries, was provided for each team. The HoNOS was the only instrument that required specific training prior to use; as this is part of the validation process (methods section) the details are not included here.

#### Data analysis

Data was collected from June through December 2001. The Statistical Package for Social Sciences (SPSS) – versions 10 – 11.5 software package was used to design the database and conduct statistical analysis; data were entered on a personal computer.

#### Statistical analysis

Paired and independent samples t-tests were first conducted to compare mean symptom counts for those with and without a PTSD diagnosis. In the sample studied there was overlap of some symptom counts for those with and without a PTSD diagnosis. In addition, for both groups, PTSD symptom counts were highly skewed. In skewed distributions measures of the mean can be relatively uninformative when used as a summary measure for comparing groups. Therefore, respondents were grouped into four subgroups: those without PTSD diagnosis whose symptom counts were in the lower 50% or upper 50% for that subset and those with PTSD diagnosis whose symptom counts were in the lower 50% or upper 50% for that subgroup. These analyses gave a more informative picture of the overall symptom presentation of those with and without a PTSD diagnosis. ANOVA were applied to this data. One sample-t-test was used for comparison with other population data. Eta-squared was used to calculate effect size in the ANOVA's.

Data relevant to outcome is based on the subjects who were still in the study at Time 2 (n = 17). Intention to treat analysis was not used as the study did not use randomised controlled methods and was an observational study only.

## Results and discussion

### Attrition

The attrition rate for the sample was 37%. Ten client/CM dyads had exited the study by the 12-month data collection point; the primary reasons for these exits were clinical and systems-based. Clinically, seven client's no longer needed case management services, therefore, were 'closed', that is, were no longer 'active' clients of the service. A further three clients changed case managers as a result of staff movements (either that of leaving the service or changing role functions) after a critical point in the research period and, therefore, exited the study as the effect for WA on outcome was not able to be measured for these clients.

### Response rate

The low responses rate of 34% was primarily a function of the large number of clients that were nominated by case managers that did not meet selection criteria. As noted in the methods section, the criterion that presented the most difficulty was that of the duration of case management service required. For the study, this was a mid- to long-term period to allow for the tracking of the therapeutic relationship (the primary variable being measured in the larger study). As clients were selected on entry to the service, CM's were required to make a judgment as to the likely term of service required. This was not always easy given the acuity of the psychopathology involved. Furthermore, in the reality of the clinical setting, the selection criteria were not uppermost in the minds of the clinicians referring the clients to the study. Attempts to address this were undertaken during the recruitment phase, for example, a large poster listing the selection criteria was designed for each team, with the request that it be prominently displayed at team meetings in which clients were allocated to case managers to aid referral. This was in addition to the planned strategies discussed in the methods section to facilitate the recruitment process. Systemic issues also impacted on the issue of eligibility as some clients referred to the study had been assigned to an interim CM only; this again made them ineligible for the purpose of measuring the therapeutic relationship as they would be changing CM's within 3 months or less.

### Data

Data on trauma/PTSD is reported for a sample group of 27 client/CM dyads (two clients withdrew their consent during baseline data collection; these data are omitted). *Where applicable, data are provided for the total sample followed by the discrete results for subgroups with PTSD (n = 9) and those reporting trauma that did not meet diagnostic threshold (n = 11) total n = 20*.

### Demographics: client

Presented in Table 1. Note that data for CM contacts and times is at 12 months and therefore only includes data for the 17 clients remaining at this point.

**Table 1:** Client Demographic details for the whole sample in sub-groupings for trauma/PTSD (n = 27)

**Variable**	**PTSD n = 9**	**Trauma/no PTSD n = 11**	**No Lifetime Trauma n = 7**
Gender M/F	5/4	5/6	4/3

Mean Age	34(SD = 12.2)	39(SD = 11)	37(SD = 14)

Country of Birth			
Australia	5	9	6
Indigenous	0	0	0
Overseas	4	2	1

Highest educational level			
Completed Secondary -Junior Level	5	4	4
Completed Secondary- Senior Level	3	6	1
Tertiary	1	1	2

Marital status			
Single never married	7	7	4
Married	1	2	1
Divorced/Separated	1	2	1
Widowed	0	0	1

Employment Status			
Employed	3	0	1
Unemployed	5	11	4
Student	1	0	0
Homemaker	0	0	2

Main Source of Income			
Government Payment	8	11	6
Private	1	0	1

Primary Diagnostic Profile			
Schizophrenia – various types	4	7	5
Borderline Personality Disorder	4	1	0
Depression	1	1	1
Bipolar Affective Disorder	0	2	1

Mean age of onset for primary diagnosis	25(SD = 12.5)	25(SD = 11.2)	25 (SD= 7.9)

Dual Diagnosis (co-occurrence substance misuse)	7	5	1

Mean number years with Tx service	5 (SD= 3.4)	8 (SD= 3.2)	8 (SD= 2.6)

Persons subject to involuntary mental health order at time of study	0	1	3

Mean number recorded contacts with CM over 12 months	29 (SD = 7) n = 5	21 (SD7.5) n = 7	33 (9) n = 5

Mean time in hours of contacts	17.5(SD= 6.5)	13(SD = 6.3)	14.6(SD = 3.2)

**Table 2:** Categories of traumatic events from the PDS screen as reported by clients (n = 20)

**TRAUMATIC EVENT**	**PTSD N = 9**		**NO PTSD N = 11**	
	**No.**	**%**	**No.**	**%**

Serious Accident, fire or explosion	7	78%	4	36%
Natural disaster	1	11%	0	
Non-sexual assault – family member or someone known	6	67%	4	36%
Non-sexual assault – stranger	3	33%	3	27%
Sexual assault – family member or someone known	5	55.5%	3	27%
Sexual assault – stranger	3	33%	2	18%
Military combat/war zone	1	11%	1	9%
Sexual contact under 18 years with someone 5 or more years older	7	78%	4	36%
Imprisonment	3	33%	2	18%
Torture	1	11%	0	
Life-threatening Illness	4	44%	2	18%
Other type of event	4	44%	7	63%

#### Co-Morbidity levels

Co-morbidity was defined as *at least *one other diagnosis (excluding PTSD). The rate of co-morbidity in the whole sample (n = 27) was 30% (n = 7) with the majority having one other diagnosis; **PTSD group n = 9: **44% (n = 4) all of whom had at least two other diagnoses; **non-PTSD group n = 11: **17% (n = 3) all of whom had only one other diagnosis.

### Demographics: case manager (n = 17)

Data is adjusted for CM's with multiple clients in the study. The majority of CM's were male (71% n = 12). The mean age was 42 years (SD= 10) range 35. The respondents had a mean of 4 years (SD = 1.2) clinical experience plus a mean of 2.5 years in mental health (SD = 1.4). Nurses comprised the largest professional discipline (71% n = 12). The remainder were Clinical Psychologists (4) and one Social Worker. The majority of CM'S had an undergraduate qualification only (59%), four had a specialised qualification in psychiatric nursing and three CM's had a higher degree at the masters' level. Fifty-three percent of respondents had post-graduate training in a related field (CBT, Counselling, Psychoeducation).

### Discussion: client demographics

#### Levels of employment

These are high at 70% in this sample, despite the high level of respondents who completed high school. The unemployment rate in the whole of the adult service of the ACT CMHS is recorded as 34%, however, this is not adjusted for acuity or diagnosis, therefore, as the sample has a high percentage of clients with a psychotic-based illness, direct comparison cannot be made.

The high educational level reported may reflect the general profile for the ACT, which has a higher level of retention of students in secondary school with 89% of year 7 students remaining in school at year 12 compared to the national rate of 73% [29]. However, place of schooling was not a demographic that was included in the screen, therefore, this is not a definitive explanation of reported educational levels in the sample.

Australia has six state and two territory governments and a federal level government, with differing levels of political responsibility for services. That of health service delivery rests at the state and territory level, and there is currently no central data collection point for employment status for persons with a mental illness in a treatment context. However, two national surveys [39,40] provide some insight into this, each reporting higher rates on unemployment (approximately 30%) across genders for persons with a mental illness. A further survey on employment in persons with psychosis [41] reported that 85% (n = 980) had their main source of income from government payments. The general unemployment rate for the ACT is 5% compared to that of 6% nationally [29].

The issue of unemployment in person with a mental illness in Australia is complex, and influenced by several factors such as access to employment-related services, stigma amongst employers and society generally, and systemic issues such as availability of vocational and rehabilitation services in mental health service delivery systems. Underpinning all of these factors is the influence of a federally-funded social welfare system that supports unemployed persons to varying degrees [29,39-41].

#### Primary psychiatric diagnosis

This was the major differentiating demographic factor between the two groups, with a greater number of clients in the non-PTSD groups diagnosed with schizophrenia. Whilst Mueser [6] found that diagnosis was the only demographic variable associated with a diagnosis of PTSD, the current sample is too small to draw any conclusions. Given the small body of research in this setting, it is difficult to be definitive about associations between comorbid psychiatric diagnoses and PTSD. As discussed below, there are many complex issues involved in any attempts to infer causality between trauma/PTSD and the development and course of other mental illnesses [8,16] and the area requires greater research.

### Data: trauma/PTSD profile

#### Rates of trauma and PTSD n = 27

Twenty clients (74%) reported exposure to at least one traumatic event; seven clients (26%) reported no experience of a traumatic event in their lifetime. Sixty-seven percent of respondents identified multiple traumatic events; two reported exposure to a single event only. The mean number of events reported was four (SD = 2.3). Nine clients from the total sample (33%) met diagnostic criteria for PTSD. Eleven clients (41%) reported trauma symptomatology that did not meet diagnostic threshold for current PTSD. Only one patient had a formal diagnosis of PTSD in their medical record.

##### Please note

The data pertinent to trauma symptomatology detailed below relates to the 20 persons who reported experiencing a traumatic event in their lifetime, this includes those meeting diagnostic criteria for PTSD (n = 9), and those reporting trauma but who did not meet diagnostic criteria for PTSD (n = 11). *The 7 clients who did not report any lifetime trauma are excluded from this section, but are included in the subsequent outcome section*.

#### Number of events (n = 20)

The total number of traumatic events reported by clients was 77; the CM reported a total of 21. Paired samples t-test showed significant difference for the mean number of reported events between CM *M = 1.0, SD = 1.12 and Clients M = 3.8, SD = 2.4 t (19), p*< .*0005*.

##### PTSD group (n = 9)

clients reported a total of 45 traumatic events (M = 5, SD = 2.95); the CM reported a total of 14 (M = 1.55, SD = 1.13) or 31 % of the events reported by the patient. The majority of CM's (78% (7)) – knew about the patient's exposure to trauma; two CM's (22%) reported no knowledge of the patient's trauma. Only one CM reported that the client was receiving treatment for trauma/PTSD.

##### Non- PTSD group (n = 11)

clients reported a total of 32 events (M = 3, SD = 1.30); CM's reported a total of 6 (M = .54, SD = .93) events or 19% of those reported by the client. The majority of CM's 64% (7) had no knowledge of the client's reported exposure to trauma.

#### Type of event

Table 2 shows the reported events. The three most frequently were *Serious Accident, Physical Assault and Sexual Assault*, however, the combination of the different types of sexual assault (childhood and adulthood) makes this the most frequently reported type of trauma across the groups. Fifty-five percent (11) of clients reported sexual assault before the age of eighteen, of these 58% (7) also reported adult sexual assault.

**Table 3:** Itemised PTSD symptoms for Criteria B, C, D, reported at 2–5 times per week

**SYMPTOM CLUSTER**	**PTSD GROUP N = 9**	**NON-PTSD GROUP N = 11**
**B: Re-experiencing**		
Upsetting thoughts or images	55.5% (5)	9.1 %(1)
Bad dreams or nightmares	55.5% (5)	0
Reliving the traumatic event	44.4% (4)	18.2% (2)
Feeling emotionally upset when reminded of the event	66.6% (6)	36.4% (4)
Experiencing physical reactions when reminded of the event	33.3% (3)	27.3% (3)
**C: Avoidance**		
Trying not to think, talk, or have feelings about the event	66.6% (6)	9.1 %(1)
Trying to avoid activities, places or people that recall the event	55.5% (5)	0
Unable to remember important part of the event	11.1%(1)	9.1 %(1)
Having less interest in important activities	66.6% (6)	9.1 %(1)
Feeling distant or cut-off from people	77.8% (7)	36.4% (4)
Feeling emotionally numb	44.4% (4)	27.3% (3)
Feeling that future plans will not come true	44.40/0 (4)	27.3% (3)
**D: Arousal**		
Having trouble sleeping	33.3% (3)	18.2% (2)
Feeling irritable	66.6% (6)	27.3% (3)
Having trouble concentrating	55.5% (5)	27.3% (3)
Being overly alert	55.5% (5)	27.3% (3)
Being jumpy or easily startled	44.40/0 (4)	9.1 %(1)

**Table 4:** Number and severity of symptoms for the 3 clusters – Median-split

**CLUSTER ITEM**		**NUMBER IN**		**MEAN**	**SD**	P*
					
		**Lower 50%**	**Upper 50%**			
	**PTSD**					
**Re-experiencing (B)**						
Number of symptoms Md: ≤ 3	***Yes***	*2*	*7*	***3.67***	*1.41*	
	No	6	5	2.18	1.94	0.072
Severity score Md: 4.5	***Yes***	*3*	*6*	***7.11***	*4.43*	
	No	*7*	4	3.36	3.04	**0.038**
**Avoidance (C)**						
Number of symptoms Md: 3.5	***Yes***	*2*	*7*	***5.11***	*1.36*	
	No	8	3	3.00	2.10	**0.018**
Severity score Md: 6.5	***Yes***	*3*	*6*	***10.78***	*5.43*	
	No	7	4	4.82	4.19	**0.013**
**Hyper-arousal (D)**						
Number of symptoms Md: ≤ 3	***Yes***	*5*	*4*	***3.78***	*1.20*	
	No	8	3	2.27	1.74	**0.041**
Severity score Md: 4.0	***Yes***	*4*	*5*	***7.78***	*4.66*	
	No	6	5	4.60	3.66	0.115
**Total number of symptoms endorsed Md: <14.5**	***Yes***	*2*	*7*	***12.67***	*3.39*	
	No	8	3	7.18	5.72	**0.021**
**Total symptom severity score Md: 9.5**	***Yes***	*3*	*6*	***25.67***	*13.77*	
	No	7	4	12.36	9.60	**0.020**

* Derived from ANOVA analysis

##### PTSD group n = 9

78% (7) of the respondents reported childhood sexual assault; 53% (5) also reported adult sexual assault (3 females, 2 males). **Non- PTSD group n = 11: **36% (4) reported childhood sexual assault; of these, 50% (2) also reported adult sexual assault (1 female, 1 male).

#### Trauma with the most effect

Interpersonal assault, physical and sexual, was the type of trauma nominated by 45% (9) of respondents as the type of trauma that 'bothered them the most'. The second highest rating trauma nominated was that of a 'serious accident or explosion' the remaining categories were unequally distributed amongst the remaining types of trauma. Eleven clients (55% n = 20) reported that the event nominated by them as the one that 'bothered them the most' happened more than 5 years ago.

### Symptom profile

Symptom details for the B C D criteria are shown in tables 3 and 4 .

#### Symptomatology specifiers (E criterion)

All clients in both groups met the **chronic symptom duration **criteria (all had experienced the symptoms for more than 3 months). **PTSD group n = 9: **The majority of clients (89% (8)) in this group had 'acute' onset of symptoms, with only one client showing 'delayed' onset. **Non- PTSD group n = 11**: In contrast, these clients were almost equally divided between the acute and delayed onset categories.

### Impairment of functioning (F criterion)

#### PTSD group n = 9

The majority of respondents – 67% (6) – met the 'severe' impairment criteria (7–9 functional areas of life effected). Two respondents met the criterion for 'moderate' impairment (3–6 areas effected); and one demonstrated 'mild' impairment (1–2 areas effected).

#### Non- PTSD group n = 11

Forty-five percent of respondents (5) showed no impairment; thirty-six percent (4) met the 'severe impairment' criteria, with one respondent in each of the remaining categories. Independent samples t-test showed significant difference in mean levels of impairment between the two groups. **PTSD **(M = 8, SD = 2.3), **non-PTSD **(M = 4, SD = 4), t (16) 2.8, p.014). Eta squared = 0.30 indicating a large effect size (Cohen).

### Discussion: trauma/PTSD profile

Findings from this profile have particular importance for CMHS and underscore the importance of trauma assessment as a routine part of entry assessment to the service as indicated below.

#### Chronicity of trauma

Despite the lengthy service contact and the historical nature of the trauma, PTSD symptomatology was still current and largely unknown to treating clinicians and was, therefore, chronic in nature. Chronicity of PTSD is associated with co-morbidity; the longer the history of PTSD, the greater the chance of an individual developing a comorbid disorder [16]. This finding highlights the importance of trauma assessment in clients with major mental illness and the hidden impact that undiagnosed and untreated trauma/PTSD may have on the course and treatment of comorbid psychiatric illness [16]

#### Multi-traumatisation

This phenomenon (another feature of PTSD) is also evident in the current study, with most clients in the PTSD group demonstrating double the trauma exposure to those of the non-PTSD Group. This finding is particularly relevant to clinicians in CMHS as multiplicity of trauma exposure is also predictive of PTSD within general and psychiatric populations [3,8]. Ipso facto, clients with multiple trauma experiences are more likely to have PTSD; therefore, screening for trauma is important in alerting clinicians to the possibility that PTSD symptomatology may be present in clients being treated for another primary psychiatric diagnosis.

#### Types of traumatic events

The pattern of events reported, although consistent with that of other studies, is quite different to that of the Australian population as reported in the findings from the NSMHW [28]; the top three categories of events in that study were 'witnessing someone being killed', 'being involved in a life-threatening accident' and 'being involved in a natural disaster'. Sexual assault (defined as rape or sexual molestation in the above survey) was comparatively small – approximately 12% (n = 10 641). These findings distinguish the 'uniqueness' of the trauma profiles in the different populations and underscore the potential reasons for the increased levels of PTSD seen in treatment populations in CMHS as discussed in a subsequent section of this article.

#### Symptom profile

The currency of the symptomatology and impairment reported as arising from the traumatic event is of interest given the chronicity of the trauma experienced. The data in table 3 showed that in the PTSD group in particular, the reports of symptomatology are clearly not an aberration or a 'one-off' experience, but a persistent experience of negative feelings and emotions associated with the trauma. Again, this is an important finding given that the trauma was largely unknown to health professionals and therefore, untreated.

Also of interest is the symptom cluster showing the greatest effect in the PTSD group -that of the 'Avoidance/Numbing' criterion. Breslau [42] notes that this has previously been the least met criterion in the PTSD symptomatology clusters and, therefore, the most critical to the diagnosis.

This cluster is also of particular interest for its interaction with other psychiatric symptomatology. As noted earlier, the predominant type of trauma experienced by clients with mental illness is inter-personal in nature, therefore, the avoidance of social interactions and feelings of detachment, feature large in this criterion, (as evidenced by the data), consequently, they have a high potential to lead to social isolation and reduced social networks. Social isolation and poor social networks are also a feature of several types of other mental illnesses and are a known predictor of relapse and hospitalisation [43,44]. Ipso, facto, co-morbid PTSD symptomatology, particularly, that of the avoidance cluster, may increase this effect and lead to a worsening of symptoms and functioning and, ultimately, relapse and hospitalisation. The hypothesised pathway by which this occurs is illustrated in the model discussed earlier [8].

#### Rates of PTSD

This finding is of major importance as it demonstrates a rate 26-times greater than that found in the National Survey of Mental Health and Wellbeing (NSMHWB) [28] (using the most conservative results for rates of PTSD) and it is also consistent with findings of other studies in persons with a major mental illness in treatment settings as discussed above. If this finding was representative of CMHS Australia wide, one third of all clients in a service at a given time may have current PTSD symptomatology. There is no reason to suspect that these findings are unique to the study site, as it does not differ greatly in structure, services or client base to other services nationwide.

### Potential contributory factors to the higher rates of trauma and PTSD found in clients of mainstream psychiatric services

The explanation for the finding of higher rates of trauma and PTSD in persons with major mental illness is unclear, however, it is evident from the discussion in the literature that this phenomena is not unique to the current study. Several potential factors (in addition to the role of chronicity discussed earlier) including victimisation, associations between age and type of trauma experienced, gender issues and vulnerability, substance use and the psychiatric setting have been proposed in the literature as possible explanations for the increased rates of trauma and PTSD in this population and these are briefly outlined below. In general these factors cannot be directly examined in relation to the results of the current study due to methodological limitations, but they provide a basis for exploration of this variable in future research studies in this population.

#### Age and type of trauma

The age at which victimisation occurs may influence later victimisation; several studies have noted the link between childhood sexual abuse and sexual and physical abuse in adulthood [3,6,45,46].

The additional relevance of this finding to general psychiatry is that sexual victimisation in childhood is also associated with the development of psychiatric disorders (other than PTSD) in adulthood [8,45,46]. The current study showed a large percentage of respondents reporting sexual abuse in both childhood and adulthood; victims of such trauma are more likely to develop other psychiatric disorders and be treated for such in the mental health system, therefore, treating health professionals need to be alert to this potential link to an unknown trauma history.

#### Gender effects

It has been suggested that women who have been subjected to sexual victimisation may be less aware of inherent dangers and have poorer risk recognition, therefore, are slower to remove themselves from sexually dangerous situations than are women with no history of sexual victimisation [8,42,47], hence increasing their vulnerability to further traumatisation. Whilst this effect is greater in some women with a history of sexual victimisation and PTSD symptomatology, this influence may be concomitant with the severity of PTSD symptoms. Better risk-awareness was reported in women with greater symptom severity, particularly those of the hyper-arousal cluster, so that PTSD symptomatology may, in some instances, act as a 'buffer' for women in sexually dangerous situations as a result of increased sensitivity to cues [47]. However, the opposite may be true for women with major mental illness, particularly those with a diagnosis of schizophrenia, who have experienced sexual victimisation. Vulnerability to re-victimisation may be exacerbated for these women as a result of the negative effect of their illness that may further compromise their social competence and decrease their ability to act positively to avert the risk or remove themselves from dangerous situations [8,48]. Sample size prohibits analysis of this factor in the current sample.

#### The Psychiatric setting

Vulnerability issues related to assaultive violence may also be a factor inherent in the psychiatric setting [20,49]; in-patient units in particular, have been the subject of a broad range of studies in relation to patient violence and the use of restraint and seclusion. However, the focus of these studies has primarily been staff and patient safety issues, the aetiology of violence, measurement issues, staff training and legislation. Even though patient to patient assault may occur, particularly in mixed gender units, few studies have examined the psychological impact of this on the individual [20]. It has been suggested that the procedures and processes of in-patient units, particularly those related to restraint and seclusion, may also re-traumatise victims [20,49]. Additionally, the experience of the mental illness itself, particularly if it involves psychosis, can result in PTSD symptomatology and/or exacerbation of previous trauma [6,49,50]. Several clients in the current study nominated this latter stressor (being diagnosed with a mental illness, particularly psychotic-based) as a 'traumatic event'. However, as it did not meet *DSM IV *criteria for a traumatic event, further assessment was not undertaken.

#### Substance use and misuse

This is a known clinical correlate of both PTSD and major mental illness, and may also play a role in the higher levels of trauma evidenced in this population [5,6,51]. The decreased inhibitory effects and resultant risk taking associated with substance abuse may place the person in increasingly unsafe social and physical environments exposing them to greater risks of interpersonal violence [6,51]. Levels of substance misuse are high in the PTSD and trauma groups of the current study, but again, the sample size prohibits any definitive analysis.

### Implications of these contributing factors for CMHS and mainstream psychiatry

The variety of issues contributing to increased rates of trauma and PTSD identified in the preceding discussion may be distilled into three main factors all of which have important implications for public-sector service providers in mainstream psychiatry, particularly CMHS. These factors relate to the *discrete sub-group *of persons accessing mainstream psychiatric services, the *inherent risk factors *for PTSD within this group and the *trauma characteristics *demonstrated by this group. Increased awareness of these factors by service providers and clinicians is the first step in responding to the identified need for service provision for clients with co-morbid PTSD.

**First**, the nature of the population; all reported studies of PTSD in persons with a mental illness are from a treatment population of persons receiving current intervention for another mental health disorder. Given the known co-morbidity associated with PTSD, it is not unreasonable that higher rates would be found in this group of persons as they are suffering non-diagnosed PTSD, of chronic duration and, therefore, the likelihood of another mental disorder developing, for which the individual seeks treatment is increased. However, awareness of this factor by service providers not only gives a contextual awareness for interpretation of research findings in the field, but also provides them with insight into the potential service needs of their customer base and the need to include trauma/PTSD screening as a routine element of entry assessment protocols in this consumer group.

**Second**, the trauma profile of persons with a major mental illness (as reported in the literature and the results of this study) is dominated by interpersonal violence, particularly sexual victimisation and is potentially a major contributing factor to the higher rates found. Whilst women in particular are at greater risk of assaultive violence [42], the type of the trauma experienced and the individual's perception of the trauma as 'upsetting' is known to influence the development of PTSD. This cognitive/emotional response is a known feature of sexual victimisation; for example, rape is one such event that is perceived as 'upsetting' across genders and in treatment and non-treatment populations and, as such, is strongly linked to the subsequent development of PTSD [3,6,28]. Whilst this underscores the need for trauma screening, it also highlights the need for a sensitive and supportive environment that allows the traumatised individual to verbalise the nature of the abuse.

**Finally**, whilst increased risk of PTSD following exposure to trauma in persons with a major mental illness is reported in both treatment and population surveys [6,15,52], this risk may be incremental depending on the type and severity of mental illness experienced; in a treatment population, the nature of the illness is likely to be more acute and/or severe, therefore, individuals may be subject to greater vulnerability to the risk factors discussed earlier, including re-traumatisation and subsequent development of PTSD.

This has particular implications for the processes and procedures associated with issues of admission and management practices in hospital units, involuntary processes, the experience of mental illness itself, particular psychotic-based illness, and the impact this has on the psychological integrity of the individual. It also highlights the need for inclusive and collaborative management of substance abuse issues and those associated with residential and environmental safety in community based services.

### The interaction of PTSD and other mental illness

#### Co-morbidity and PTSD

PTSD is strongly co-morbid with other psychiatric disorders as demonstrated in the two community surveys in America [3] and Australia [2]. The NSMHWB [2] found a 12-month prevalence of co-morbidity for PTSD with at least one other Axis 1 diagnosis in 85.2% of males and 79.7% of females, whilst the National Co-morbidity survey showed 88.3% males and 79% females with at least one other psychiatric disorder [3]. This high rate of co-morbidity was also demonstrated in the current study particularly in the PTSD group, who accounted for most of the co-morbidity in the sample, however, the sample was too small to examine gender differences.

Notwithstanding the potential influence of the above on rates of PTSD in persons with a co-morbid psychiatric diagnosis, no definitive causality chain between PTSD and other mental disorders can be identified. Although some researchers discuss the seemingly intuitive link between traumatic experiences, PTSD and the course of other mental illnesses (based on the widespread occurrence of the phenomenon), there is no definitive answer to the question at this time and the issue has not been widely studied or reported.

The possible link between the two broad types of disorders may be more to do with shared risk factors for PTSD and other mental health problems, such as the mood disorders, and the interplay between differential effects of specific traumas, individual risk factors and personal coping mechanisms [16,28]. The full explanation may lie in a complex matrix of all of these factors differing across individuals, communities and diagnoses, but is unlikely to lie in the potential for symptom overlap between PTSD and common co-morbid diagnoses such as depression, anxiety, and dysthymia. Rather, the literature suggests that this factor, far from overstating the case for PTSD, may result in misdiagnosis if detailed trauma histories are not sought and potential PTSD diagnosis excluded [10,15,16].

The potential influence of this factor cannot be ruled out of the current study given the level of documentation of respondents' trauma/PTSD profile found. Although this local finding was not unexpected, as there was no formal or standardised assessment of trauma done in the service it does, however, demonstrate the potential for diagnostic ambiguity in clients presenting with symptoms of depression, anxiety, psychosis and/or substance abuse, if trauma histories are not routinely sought.

Whilst the inter-relationship between trauma, PTSD and other mental illness is complex and largely unexamined, thus compounding attempts to explain the higher rates found in persons with other mental disorders, contemporary findings indicate that multiple traumatisation is a strong predictor of PTSD in treatment and non treatment populations regardless of the ultimate relationship [3,6]. The single definitive answer that can be gleaned from the current findings and discussions on the subject, is that much more rigorous and longitudinal research is needed from an epidemiological and targeted perspective in order to achieve optimal outcomes for the commonly treated psychiatric disorders [1,3,7,8,16,22,28,45,53,54].

### Data: effect of PTSD diagnosis on client outcome

As the study sought to explore the potential effect of untreated PTSD symptomatology on client health outcomes, this section compares data for clients with PTSD to those without PTSD, and therefore, includes those clients who reported no experience of trauma either current or lifetime. The sample size was 17 (5 clients with PTSD, 12 without), the number remaining enrolled in the study at the T2 data point, the 6-month period following engagement with the service and CM. Only two of the four outcome measures used showed any significant difference for clients with PTSD – the HoNOS and the WHOQOL. Only one of these, WHOQOL, is reported in this paper for reasons discussed earlier. Data are presented in table 5/6. Two population 'norms' were used for comparison, the first was from a similar population of clients with a major mental disorder, primarily Axis 1 diagnoses, 70% of whom had a diagnosis of Schizophrenia [55]. That study was conducted in an Australian Community Mental Health setting and used the WHOQOL-BREF, client and CM format and, therefore, provides a suitable comparison in view of the lack of studies in clients with major mental illness and PTSD. The second comparison is that of the Australian population 'norms' for the WHOQOL [33].

**Table 5:** QOL PTSD/No PTSD diagnosis (n = 17): Client data and comparisons with community samples.

**Domain**	**Study Group: Client Data**				**Population 'Norms' Psychosis n = 173**			**Australian Population n = 396**		
	**M**	**SD**	**P**	**Effect size (Eta^2^)**	**M**	**SD**	P*	**M**	**SD**	P*

**Physiological Health**					60.7	15.4		80	17.1	
***PTSD n = 5***	**37.14**	8.60	**.034**	0.26			**.004**			**.0005**
No PTSD n = 12	59.22	20.17					.805			**.004**
**Psychological Health**					56.8	17.4		72.6	14.2	
***PTSD***	**32.50**	9.50	**.010**	0.36			**.005**			**.001**
No PTSD	58.33	18.37					.778			.021
**Social Relationships**					51.3	20.3		72.2	18.5	
***PTSD***	**38.33**	26.74	.527				.339			**.047**
No PTSD	45.13	16.46					.221			**.0005**
**Environment**					61.1	13.8		74.8	13.7	
***PTSD***	**50.62**	10.22	**.015**	0.33			.084			**.006**
No PTSD	69.72	13.57					.061			.186

Derived from one sample t-test.

**Table 6:** QOL PTSD/No PTSD diagnosis: CM data and comparisons with 'psychosis" sample

**Domain**	**Study Group: CM Data**				**Psychosis 'Norms'**		
							P*

	**M**	**SD**	**P**	**Effect size (Eta^2^)**	**M**	**SD**	

**Physiological Health**					**57**	**12.5**	**.110**
**PTSD n = 5**	**38.75**	20.10	**.001**				
No PTSD n = 12	70.23	11.82					
**Psychological Health**					**51**	**13.0**	**.032**
**PTSD**	**36.66**	9.94	**.007**				
NO PTSD	54.16	10.87					
**Social Relationships**					**43.4**	**18.8**	**.026**
**PTSD**	**26.66**	10.86	.066				
No PTSD	40.97	14.41					
**Environment**					**55.4**	**13.5**	**.178**
**PTSD**	**46.87**	11.69	**.006**				
No PTSD	65.62	10.74					

There were no significant correlations between client and CM ratings on any of the outcome measures used in this study.

### Discussion: effect of PTSD on client outcomes

The findings on outcome are severely constrained by the short follow-up time and no definitive conclusions can be drawn from the lack of effect for PTSD Diagnosis on the other outcomes selected.

Whilst QOL is considered the most important outcome in mental health research and is central to outcomes management [56], there is an obvious lack of literature on PTSD and quality of life in the study population with which to compare the research findings [56,57]. Contemporary QOL research in PTSD stems primarily from a veteran's perspective [56,57]; research of civilian trauma has focused on specific trauma-related perspectives, for example, female victims of violence [58], victims of major trauma [59], or persons with specific medical conditions [60,61] and those involved in drug trials [57]. Although there remains the issue of different QOL measurements used in the various studies to date, the emerging trend in anxiety research suggests that PTSD in particular, has a major negative effect on QOL [56,57].

The results of the current study support this growing body of research, with clients and CM's both reporting data that was significantly different for those clients with PTSD than those without, in three of the four domains of QOL measured.

The impact of co-morbid PTSD on QOL in persons with another major mental illness is further evidenced in the study group when compared with a similar population with major mental illness but without PTSD. Clients with PTSD showed significantly greater impairment in physiological and psychological health than did those in the comparison group who had a psychotic illness only (considered to be one of the most disabling disorders with a lower quality of life than that reported in physical illness and the general population). In contrast, those study clients without PTSD had scores very similar to that reported for the comparison group. Additionally, the study demonstrated that for clients with and without PTSD, reported QOL was significantly worse in all domains (with the exception of that of the Environmental Domain in the non-PTSD group) when compared with that of the general Australian population.

## Limitations

The primary limitations of this study were the small sample size and the self-report nature of the trauma/PTSD data collected from the client. Although an attempt to minimise this latter point was taken by conducting the PDS as an interview, clients still needed to engage in re-call, as the trauma was an historical event for most of them. Furthermore, it also required discrimination of similar symptomatology from that of their primary psychiatric diagnosis. Both of these limitations are addressed in the recommendations.

## Conclusion

The trauma/PTSD profile for this small sample of Australian CMHS clients with major mental illness was consistent with findings from other reported studies of similar populations on all key elements and was largely unknown by treating clinicians.

Standardised assessment of trauma was not a routine element of service entry at the study site or generally within Australian Community Mental Health Services, but there is growing evidence within the literature and from the current study to support the introduction of such a measure.

This study also identified poorer outcomes in clients with PTSD, albeit for a single outcome only, that of QOL, which was shown to be significantly compromised both within the study group and in comparison to that reported for an external population of clients with psychotic-based illness (no PTSD) in another CMHS within Australia in which the same measure of QOL was used. No effect was found for other key outcome measures with the exception of the HoNOS, which will be reported elsewhere.

Whilst no single explanation for the findings of increased trauma/PTSD in persons with another major mental illness was evidenced either in the current study or the literature, the authors propose that three interconnected factors, primarily related to the characteristics of the population treated in CMHS, may figure large in any such explanation. Each of these has important implications for service delivery across all elements of an integrated mental health service.

## Recommendations

Finally, within the context of these findings it is recommended that further research be undertaken with a larger sample to determine the relevance of these findings to the broader population of clients in Public Community Mental Health Services. In addition to the use of a screening measure, a follow-up clinical interview, such as the Clinician Administered Posttraumatic Stress Scale (CAPS) [62] is recommended in order to provide more robust data that clearly distinguishes PTSD symptomatology from that of the primary psychiatric disorder.

## Competing interests

The authors declare that they have no competing interests.

## Authors' contributions

IH participated in study design, data collection and analysis and drafted the manuscript. CO coordinated and facilitated the study at the clinical interface in ACT, participated in study design, and preparation of manuscript. PY conceived the study and participated in study design and implementation in Brisbane, and acted as overall consultant. LM participated in the study design and implementation in Brisbane and provided specialist advice relating to Posttraumatic Stress Disorder measures. FD participated in study design for the Brisbane elements of implementation and facilitated the study at the clinical interface. RP provided statistical advice and analysis and participated in the preparation of the manuscript. All authors read and approved the final manuscript.

## Pre-publication history

The pre-publication history for this paper can be accessed here:


